# Substance Use Disorders (SUD) and Suicidal Behaviors in Adolescents: Insights From Cross-Sectional Inpatient Study

**DOI:** 10.7759/cureus.15602

**Published:** 2021-06-11

**Authors:** Sayeda A Basith, Miles M Nakaska, Albulena Sejdiu, Aabha Shakya, Vaishalee Namdev, Siddharth Gupta, Keerthika Mathialagan, Ramkrishna Makani

**Affiliations:** 1 Psychiatry and Behavioral Sciences, Medical University of the Americas, Charlestown, KNA; 2 Family Medicine, American University of the Caribbean, Calgary, CAN; 3 Psychiatry, Cyril and Methodius University, Skopje, MKD; 4 Family Medicine, Saint James School of Medicine, Kingstown, VCT; 5 Medicine, Mahatma Gandhi Memorial Medical College, Indore, IND; 6 Internal Medicine, Sri Guru Ram Das Institute of Medical Sciences and Research, Amritsar, IND; 7 Psychiatry, Sree Balaji Medical College and Hospital, Chennai, IND; 8 Psychiatry, AtlantiCare Health System, Egg Harbor Township, USA

**Keywords:** substance recreational use, child and adolescent psychiatry, suicide risk, suicide behavior, alcohol use

## Abstract

Objectives

To determine the demographic predictors of suicidal behaviors and measure the association between the spectrum of substance use disorders (SUD) and hospitalization for suicidal behaviors in the adolescent population.

Methods

We conducted a cross-sectional study using the nationwide inpatient sample and included 466,244 adolescent inpatients with psychiatric illnesses. The study sample was sub-grouped into suicidal (N = 182,454) and non-suicidal (N = 283,790) cohorts. The odds ratio (OR) of association for demographic characteristics and comorbid SUD in the suicidal group was evaluated using a logistic regression model witha P-value < 0.01.

Results

Our study population included 466,244 adolescent inpatients. Females had higher odds of suicidal behaviors (OR 1.45; 95% CI 1.431-1.470) compared to males. The most prevalent comorbid SUD among suicidal inpatients was cannabis (15.3%) but had a statistically non-significant association with suicidal behaviors (OR 0.98; 95% CI 0.95-0.99). Inpatients with alcohol use disorders had significantly increased odds of association with suicidal behaviors (OR 1.18; CI: 1.142-1.209) compare to other SUD. Among other substances (cannabis and stimulants), there existed a statistically non-significant association with hospitalization for suicidal behaviors.

Conclusion

Adolescent inpatients with comorbid alcohol use disorders were at 18% higher odds of hospitalization for suicidal behaviors. Our study provides a basis for more research while also suggesting potential avenues for early identification and intervention efforts for comorbid SUD in adolescents requiring psychiatric care to improve their prognosis and quality of life.

## Introduction

Substance use disorders (SUD) have serious consequences for children and adolescents’ physical and mental health. The lifetime prevalence of any illicit substance use among adolescents is 42.9% [1]. The most commonly abused substance among teens is alcohol as they consume about 1/10th of all alcohol consumed in the United States (US), followed by tobacco and cannabis [2]. According to a 2019 youth risk behavior survey (YRBS) in the US, 14.8% of high school students used illicit substances, 14.3% misused prescription opioids, and 1.6% have injected illegal substances. The overall percentage of substance use has decreased among Caucasians and Hispanics, but it has remained unchanged in African Americans during this 10-year trend descriptive study. Although high-risk substance use behaviors have declined since 2009, nearly one in every seven adolescents still report drug use and misuse [3].

Several factors, including genetic predisposition, adverse familial and social environment, and substance abuse at a young age, could all contribute to addiction [4]. The “reward system” is a major pathophysiological contributor to addiction in adolescents and the adult population. The brain receives far more dopamine after a “drug high” than the natural reward system would produce [4]. This dopaminergic reward pathway acts on the nucleus accumbens, providing positive reinforcement for substance abuse. The majority of stimulants exert their effect by increasing dopamine, norepinephrine, and serotonin levels in synapses [1]. Alcohol and sedatives/hypnotics, on the other hand, depress the central nervous system arousal by enhancing the action of gamma-aminobutyric acid (GABA) [1]. Research has demonstrated that certain developmental stages of adolescence result in higher vulnerability to SUD [5]. Furthermore, early substance exposure during a period when the brain is undergoing significant developmental changes is associated with a more chronic and severe course of SUD than is seen if started in older age groups [6].

Suicide accounts for approximately six percent of all adolescent deaths worldwide, and it is the second leading cause of death among children and youth aged 10 to 24 years old [7]. According to the YRBS 2019 report, approximately nine percent of youth in grades nine to 12 attempted suicide at least once during the year [3]. Major risk factors are being female, being of African American descent, exposure to bullying and violence, sexual orientation, alcohol and substance abuse, psychiatric comorbidities, poor familial, and social support [8]. SUD often coexist with psychiatric illnesses and further increases the suicide risk [9-11]. Adolescents with SUD and suicidal behaviors share similar risk factors that may interact with each other. Past studies have found adolescents with psychiatric comorbidities like depressive disorders and attention-deficit/hyperactivity disorder were at a higher risk of SUD, which can lead to impairment of overall functioning [12,13]. Our study focuses on identifying the demographic predictors of suicidal behaviors and assessing the relationship between the spectrum of SUD and suicidal behaviors in the adolescent population.

## Materials and methods

Study sample

We conducted a cross-sectional study using the national (nationwide) inpatient sample (NIS), which is the largest inpatient database in the US. The NIS constitutes data from 4,411 non-federal community hospitals across 44 states in the US [14]. We included 466,244 adolescent inpatients (aged 12 to 18 years) with a psychiatric illness as their primary discharge diagnosis. The sample was divided into two groups: those who did not engage in suicidal behavior/non-suicidal group (N = 283,790) and those who did engage in suicidal behavior/suicidal group (N = 182,454).

Variables

The demographic variables included in this study were age at admission, sex, race, and geographic region in the US. Comorbidities are co-diagnosed conditions found in inpatient records, and we included SUD such as alcohol, cannabis, stimulants (cocaine and amphetamine), and opioids [15].

Statistical analysis

We compared the distributions of demographic characteristics and comorbid SUD in adolescent inpatients without versus with suicidal behaviors by performing descriptive statistics and Pearson’s chi-square test for categorical variable and independent sample T-test for continuous variable (age). The odds ratio (OR) of association for demographic characteristics and comorbid SUD in the suicidal group was evaluated using a binomial logistic regression model with the non-suicidal group as the reference category. All analyses were conducted with the help of a statistical package for the social sciences (SPSS) version 26.0 (IBM Corp., Armonk, NY, USA) and with statistical significance was set at a two-sided P-value < 0.01.

Ethical approval

The NIS is publicly available de-identified data with the protection of patients, physicians, and hospital-related information; henceforward, we were not required to obtain institutional review board approval for our study [14].

## Results

Our study population included 466,244 adolescent inpatients. 59.9% of our study population were females, while 40% were males. Caucasians made 62.3% of our population, and 39.1% of inpatients endorsed suicidal behaviors. A higher proportion of these inpatients with suicidal behaviors were females (65.3%) compared to 56.5% of females in the non-suicidal group. Suicidal behaviors were also more prevalent in Caucasians (65.3%), followed by blacks (14.9%), and Hispanics (12.7%). A higher proportion of adolescent inpatients with suicidal behaviors were prevalent in the midwest (37.7%) and the south (35.1%) but lower in the northeast (14.9%) and the west (12.3%) regions of the US.

The most prevalent comorbid SUD among suicidal inpatients was cannabis (15.3%), but it was significantly lower than that seen in the 16.4% non-suicidal group (P < 0.001). Approximately 6.6% of inpatients with suicidal behaviors had comorbid alcohol use, which was significantly higher than the non-suicidal cohort (six percent). Opioid use (2.6%) and stimulant use (2.4%) were observed in a higher proportion of the non-suicidal cohort as shown in Table 1.

**Table 1:** Descriptive characteristics of the study sample SD: standard deviation

Demographic	Suicidal (no)	Suicidal (yes)	P-value
Number of inpatients	283,790	182,454	-
Mean age (SD), in years	15.4 (1.9)	15.3 (1.8)	0.545
Sex, in %			
Male	43.5	34.7	<0.001
Female	56.5	65.3	
Race, in %			
Caucasian	60.4	65.3	<0.001
Black	18.6	14.9	
Hispanic	13.3	12.7	
Others	7.8	7.1	
Region, in %			
Northeast	23.1	14.9	<0.001
Midwest	28.5	37.7	
South	36.2	35.1	
West	12.2	12.3	
Comorbid substance use disorders			
Alcohol use disorder	6.0	6.6	<0.001
Cannabis use disorder	16.4	15.3	<0.001
Stimulant use disorder	2.4	1.8	<0.001
Opioid use disorder	2.6	1.6	<0.001

Females were more likely than males to be hospitalized for suicidal behaviors (OR 1.45; 95% CI 1.431-1.470). When compared with Caucasians, other races had a negative association with hospitalization for suicidal behaviors (P < 0.001). Likewise, compared to the northeast, the adolescent inpatients in the midwest (OR 1.98; 95% CI: 1.95-2.02), the west (OR 1.52; CI: 1.48-1.53), and the south (OR 1.51; CI: 1.48-1.53) had a significantly higher odds of hospitalization for suicidal behaviors.

Among SUD, only alcohol had significantly increased odds of association with hospitalization for suicidal behaviors (OR 1.18; CI: 1.142-1.209). Among other substances (cannabis and stimulants) there existed a statistically non-significant association with hospitalization for suicidal behaviors as shown in Table 2.

**Table 2:** Logistic regression analysis of odds ratio for suicidal behaviors in adolescent inpatients

Variable	Odds ratio	95% Confidence interval	P-value
Mean age	0.99	0.990–0.997	<0.001
Sex			
Male	Reference		<0.001
Female	1.45	1.431–1.470	
Race, in %			
Caucasian	Reference		<0.001
Black	0.75	0.730–0.760	
Hispanic	0.94	0.917–0.954	
Others	0.87	0.852–0.896	
Region			
Northeast	Reference		<0.001
Midwest	1.98	1.95–2.02	
South	1.51	1.48–1.53	
West	1.52	1.48–1.53	
Comorbid substance use disorders			
Alcohol use disorder	1.18	1.142–1.209	<0.001
Cannabis use disorder	0.98	0.958–0.996	0.017
Stimulant use disorder	0.92	0.855–0.982	0.013
Opioid use disorder	0.66	0.628–0.692	<0.001

## Discussion

Suicidal behavior is a significant public health issue in the adolescent populations in the US. Suicidal behaviors encompass a continuum, ranging from suicidal ideation or thoughts of suicide to planning for suicide, suicide attempts, and death by suicide [16]. The mechanisms underlying suicidal behaviors are complex and heterogeneous, with multiple etiologies [17].

Our study provided an epidemiological view of distinct at-risk subgroups within this population, as well as identified at-risk subgroups with suicide risk among inpatient adolescents. We found that suicidal behaviors are more common in white females. Evidence suggests an increased rate of depression among females leading to increased suicidality [18]. The discrepancy could be due to various biological and psychosocial disparities in females. While female counterparts experience variable adverse stressors, their temperament and response to these stressors play an integral part in the development of depression [19].

Our findings are consistent with trends that show the geographic disparity in adolescent suicidality with higher prevalence in the midwest, the south, and the western regions of the US. There exists a correlation between suicide rates, and availability and utilization of psychiatric care and barriers to access psychiatric care, and regional differences in the state mental health parity laws. Also, there exist differences in psychiatric care resources and economic differences between urban and rural populations [20].

Our study found that alcohol use disorders significantly increased the odds of hospitalization for suicidal behaviors in adolescents. These results support a previous study by Swahn and Robert that has found that preteen alcohol use initiation was associated with suicidal ideation (increased by 1.89 times) and suicide attempts (increased by 2.71 times) relative to nondrinkers for both boys and girls [21]. An increased rate of suicides is seen in the contiguous post-consumption period and long-term use of alcohol. The theory is that alcohol could induce acute aggression and impulsivity during the post-consumption period, leading to increased suicidal behaviors. And in the long term, alcohol consumption could cause biological changes and negative psychosocial events that could potentiate pre-existing issues influencing suicidal behaviors [22]. These deficits are more pronounced among youth who have used alcohol earlier in their life [23].

Although the prevalence of cannabis use disorder was highest among the suicidal inpatients, our study found no significant association between cannabis use and suicidal behaviors. A recent review and meta-analysis discovered a lack of consistent evidence linking acute cannabis use to increased risk for suicidality [24]. Evidence supports that chronic cannabis use can lead to suicidal behaviors, but the lack of systemic control for known risk factors tempers this finding. Our study demonstrated lower odds of opioid use in association with suicidal behaviors. However, as per the study by Wilkins et al., any prescription opioid misuse (current and past misuse) is associated with increased students' risk of suicide-related behaviors but our study did not determine any causal association [25].

Our study had limitations. To begin with, we used administrative data that lacked patient-level clinical information, and inpatients' diagnoses were based on the diagnostic codes, which may cause underreporting of comorbidities. Furthermore, because we conducted a cross-sectional study, we were unable to identify a causal relationship between suicidal behaviors and SUD. Despite this, the use of NIS offered an incomparable population-based perception of disease associations with systematic and temporal factors. Given the large sample size with the national representation of the population, results were appropriate for generalizability for the inpatient population.

## Conclusions

It is essential to understand the impact of SUD on suicidal behaviors among adolescents. Adolescent inpatients with comorbid alcohol use disorders were at 18% higher odds of being hospitalized for suicidal behaviors. Our study provides a basis for more research while also suggesting potential avenues for early identification and intervention efforts for comorbid SUD in adolescents requiring psychiatric care to improve their prognosis and quality of life.
